# Genome-Wide Detection of Spontaneous Chromosomal Rearrangements in Bacteria

**DOI:** 10.1371/journal.pone.0042639

**Published:** 2012-08-03

**Authors:** Song Sun, Rongqin Ke, Diarmaid Hughes, Mats Nilsson, Dan I. Andersson

**Affiliations:** 1 Department of Medical Biochemistry and Microbiology, Uppsala University, Uppsala, Sweden; 2 Department of Immunology, Genetics and Pathology, Uppsala University, Uppsala, Sweden; The Roslin Institute, University of Edinburgh, United Kingdom

## Abstract

Genome rearrangements have important effects on bacterial phenotypes and influence the evolution of bacterial genomes. Conventional strategies for characterizing rearrangements in bacterial genomes rely on comparisons of sequenced genomes from related species. However, the spectra of spontaneous rearrangements in supposedly homogenous and clonal bacterial populations are still poorly characterized. Here we used 454 pyrosequencing technology and a ‘split mapping’ computational method to identify unique junction sequences caused by spontaneous genome rearrangements in chemostat cultures of *Salmonella enterica* Var. Typhimurium LT2. We confirmed 22 unique junction sequences with a junction microhomology more than 10 bp and this led to an estimation of 51 true junction sequences, of which 28, 12 and 11 were likely to be formed by deletion, duplication and inversion events, respectively. All experimentally confirmed rearrangements had short inverted (inversions) or direct (deletions and duplications) homologous repeat sequences at the endpoints. This study demonstrates the feasibility of genome wide characterization of spontaneous genome rearrangements in bacteria and the very high steady-state frequency (20–40%) of rearrangements in bacterial populations.

## Introduction

Genome rearrangements such as duplications, deletions and inversions have important effects on bacterial gene expression and evolution, including genome reductive processes and creation of new genes. Most studies of genome rearrangements in bacteria have relied on the comparisons of closely related genomes and searches for non-syntenic chromosomal regions [1]–[3]. The comparisons can be made at different levels: interspecies (e.g. between *E. coli* and *S. enterica*), intraspecies (e.g. different serovars of *S. enterica*), between different clonal types (e.g. different clinical isolates descending from the same clone), and finally by detection of spontaneously occurring genome rearrangements (SGRs) within a growing population derived from a single or small number of cells. The major technologies used to compare bacterial genomes include physical mapping by pulsed-field gel electrophoresis (PFGE), global comparative hybridization studies using microarrays, and whole genome sequencing (WGS). In unselected bacterial populations, SGRs are not fixed and are usually present in very low frequencies. For example, even though duplications are among the most frequently occurring genome rearrangement events, in an unselected bacterial population the frequency of cells with a duplication only ranges between 10^−2^ and 10^−5^ depending on the region [4]. Consequently, none of the aforementioned technologies can be directly used to detect spontaneous genome rearrangements (SGRs), because the frequencies of SGRs are too low to generate detectable signals in PFGE or microarray based hybridization method and the technical difficulties associated with isolating and sequencing genomes of individual bacterial cells for WGS. A similar question, detecting structural variants between individual human genomes or in cancer genomes, has been extensively addressed using sequencing based methods [5]–[10]. Most recent studies have used pair-end reads for structural variants discovery, which is based on the mining of read pairs that align differently than the reference human genome. This approach requires further PCR confirmation of putative structural variants using primers spanning possible breakpoints. However, PCR is poor at detecting very rare target DNA, which is typically the case for SGRs in an unselected bacterial population. In this study we employed a new technique, padlock probes hybridization, to validate putative SGRs. This technique requires the breakpoint sequences to be determined to base pair resolution, which led us to choose “split mapping” as the computational method (described in Materials and Methods). The employed sequencing technology was 454 pyrosequencing because long read-lengths are critical in detecting reads with “split mapping” signature [11].

Using the strategy described above, we conducted a genome-wide detection of SGRs in a bacterial population that was continuously grown for 240 generations in a chemostat. Our results suggest that genome rearrangements are common in bacterial populations and that their frequencies rapidly reach steady state.

## Results

### Experimental set-up

**Figure 1:**
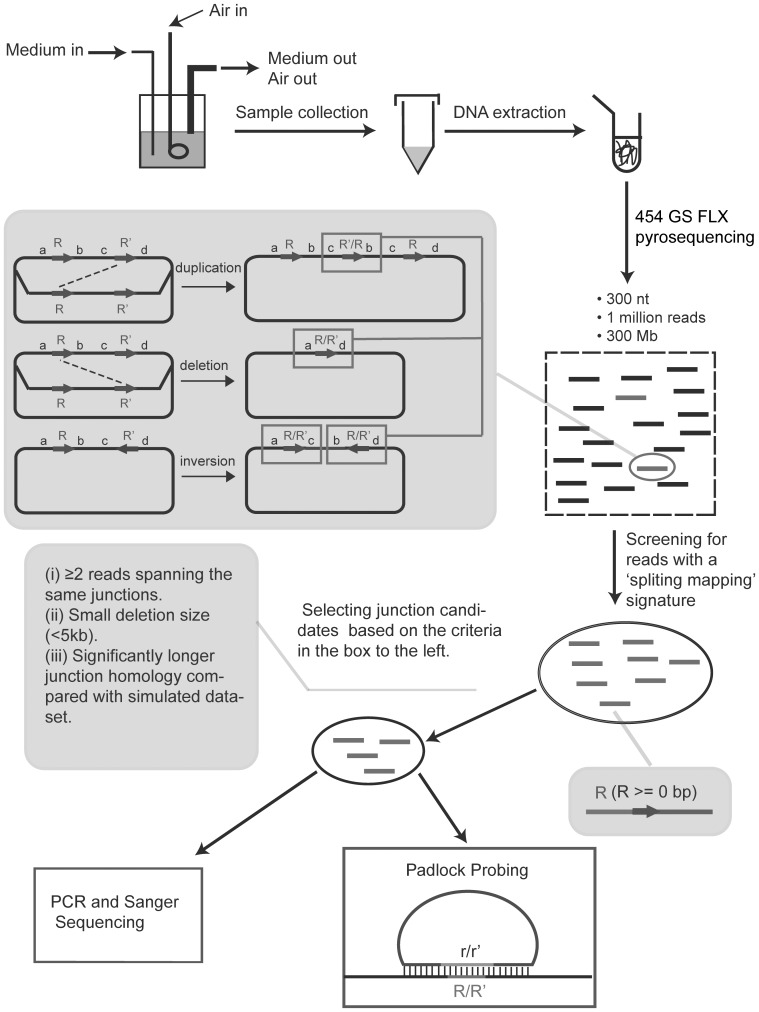
Workflow of detection and verification of SGRs in *S. typhimurium*. The SGRs detection and verification procedures in this work are as followings: (i) bacterial cells were grown in a chemostat for five days; (ii) samples were collected at each day and 454 pyrosequencing was performed on genomic DNA prepared from three samples collected at day one, two and three; (iii) reads with ‘split mapping’ signature were mined from the three datasets and further subjected to the confirmatory screening based on the three listed criteria; (iv) A substantial fraction of putative rearrangements were selected for experimental verification using padlock probe hybridization and/or PCR.

Starting from a population of <10 cells, *Salmonella enterica* Var. Typhimurium LT2 (designated as *S. typhimurium* throughout the text) was grown in a chemostat at 37°C for up to 240 generations. Bacterial cultures were subsequently collected at generation 48, 144 and 240 (designated as gen48, gen144, and gen240 throughout the text) and used to prepare total DNA for sequencing. Growing bacterial cells in a chemostat and collecting samples at three different time points allowed us to examine how fast genome rearrangements approach their steady state frequencies and inoculation with a very small population (<10 cells) avoided cells with pre-existing rearrangements in the chromosome. Genomic DNA of sample gen48, gen144 and gen240 were prepared and further sequenced on Roche/454 FLX Pyrosequencer. In total ∼1 million reads of ∼300 bases were generated and the average sequencing coverage was calculated to be 63-, 48-, and 23-fold for the three samples from gen48, gen144 and gen240 respectively. A read spanning a rearrangement junction will leave a “split mapping” (Figure S1) signature in the reference genome, with a prefix and suffix of the read mapped to different genomic locations. Reads with such ‘split mapping’ signature suggested possible rearrangements and were subjected to the confirmatory screening based on the three criteria described in Materials and Methods. A substantial fraction of putative rearrangements were further verified by padlock probe hybridization and/or PCR (Figure 1).

### Classification of putative rearrangements

The relative chromosomal orientation and location of the prefix and suffix in a read sampled across a putative rearrangement were used to classify a putative rearrangement (Figure S1). The classification system used in this study is described as: rearrangements are classified as (1) inversions, if the two split segments were mapped in different orientations; (2) deletions or duplications, if the two split segments were mapped in the same orientation. In the latter case, it is technically difficult to distinguish between deletions and duplications due to the circularity of the bacterial chromosome. However, one could infer the likelihood of a putative rearrangement being a deletion or duplication on the basis of the possible deletion or duplication size. Thus, if the split distance (from the prefix to the suffix along the mapped orientation) is very small (e.g. several kb), this suggests either a small deletion or a duplication that almost duplicates the whole genome. It is more likely to be the former one because spontaneously occurring whole genome duplications are expected to be rare in bacterial populations given the observed variation of the frequencies of spontaneous duplications between different chromosomal locations [4]. On the other hand, if the split distance is large, this suggests either a large deletion or a relatively small duplication, the duplication would be favored because large deletions are likely to be lethal. Therefore, for the purposes of this report deletion-or-duplication rearrangements (rearrangements with two split segments mapped in the same orientation) are further classified as deletions, if the split distance is ≤5 kb, or duplications, if the split distance is >5 kb. The distance threshold (5 kb) used for this second-step classification is based on the observed size distribution of putative rearrangements identified from this work, which will be discussed in more detail later in this paper. A schematic representation of how deletion, duplication and inversion could be formed is given in Figure 1.

### Detection of putative rearrangements

**Figure 2:**
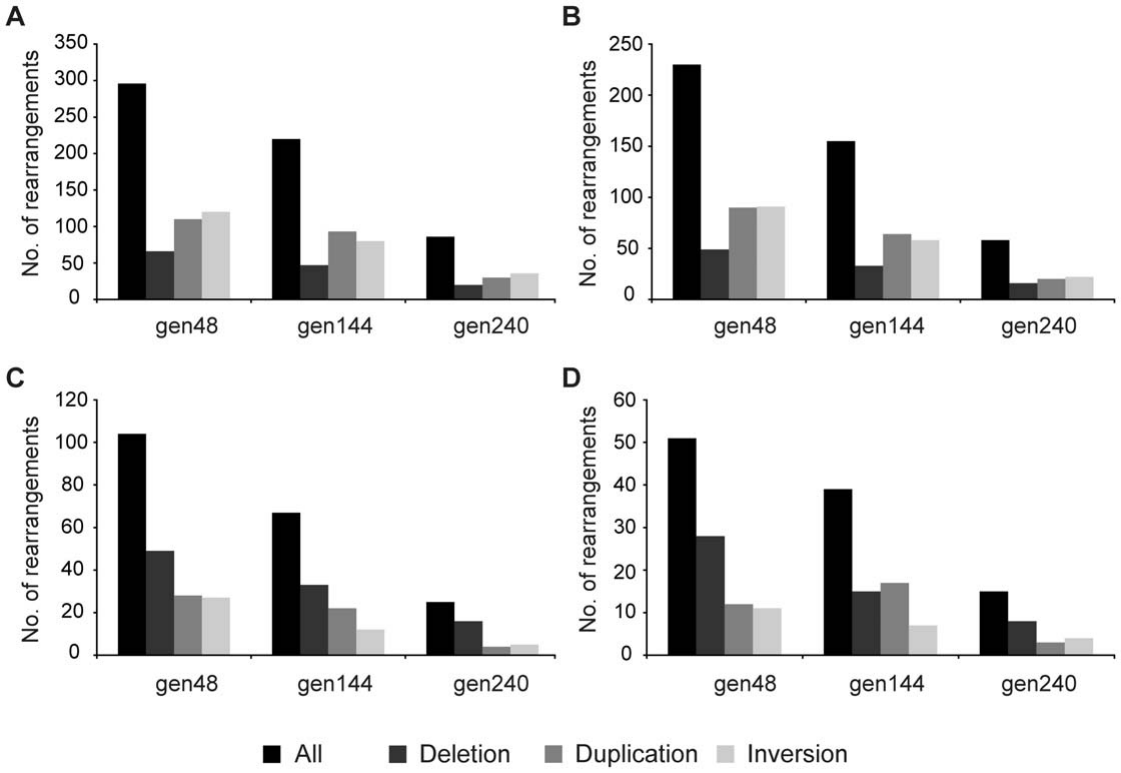
Summary of stepwise detection and verification of genome rearrangements. (A) After initial screening based on the ‘split mapping’ signatures (B) After removal of artifacts and quality score analysis (C) After confirmatory screening based on the three criteria (D) After Padlock Probe and PCR verification (expected true rearrangements).

**Figure 3:**
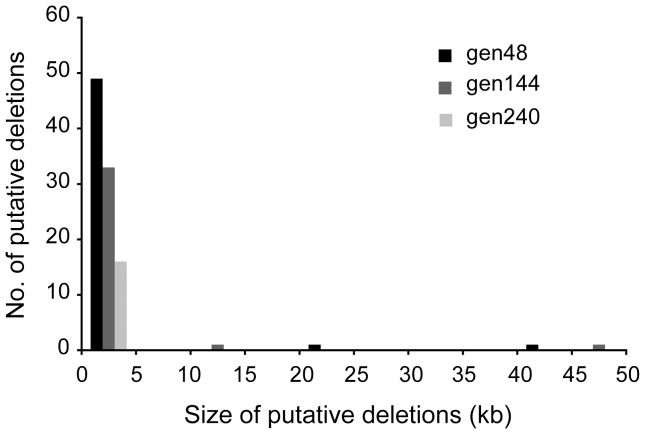
Size distribution of putative deletions. The size distribution of putative deletions with sizes less than 50 kb was examined for the three datasets, gen48, gen144 and gen240.

**Figure 4:**
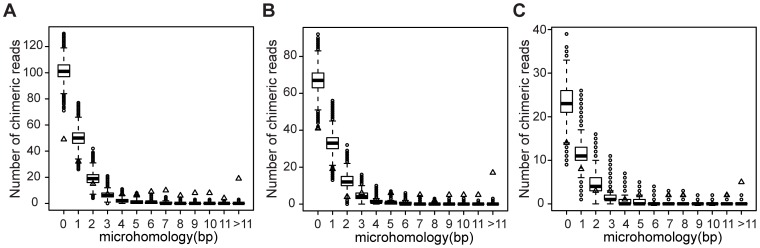
Junction microhomology analysis. The distribution of overlapping microhomologies at junctions was compared between the three datasets (gen48, gen144 and gen240) and one simulated dataset using 20 million in silico chimeric reads. The observed junction microhomology distribution in the datasets gen48, gen144 and gen 240 were represented by triangles and the simulated distributions were represented by boxplot. (A) Comparison between the dataset gen48 and the simulated dataset (181 rearrangements). (B) Comparison between the dataset gen144 and the simulated dataset (120 rearrangements). (C) Comparison between the dataset gen240 and the simulated dataset (42 rearrangements).

After the initial screening, there were 296 reads from gen48, 220 from gen144 and 86 from gen240 in which the “split mapping” signature suggested a unique putative rearrangement (Figure 2A). The number of reads suggesting possible rearrangements were reduced to 230, 155 and 58 for the three datasets, gen48, gen144 and gen240, respectively after examination of the quality scores of bases at junctions as described in Materials and Methods (Figure 2B and Table S1). These candidate rearrangements were then judged based on the three criteria listed in Materials and Methods. The first criterion is that if there are ≥2 reads that span the same rearrangement junction it is likely to be true, as it is highly unlikely that exactly the same rearrangement junction is artefactually generated. Thus, repeated findings of the same junction provide strong indications for the occurrence of the same SGRs in the bacterial population. However, since each individual SGR is generally quite rare in the population it is expected that the occurrence of ≥2 reads spanning the same rearrangement junction should be uncommon. Indeed, only three, five and one putative rearrangements were identified based on the first criterion in the three datasets gen48, gen144 and gen240 respectively and the majority of reads with the “split mapping” signature were singletons (Table S1). During the library preparation for 454 pyrosequencing, when the DNA fragments were ligated to the adaptors, chimeras could be possibly formed by ligations between concatenated fragments and the adaptors. These chimeric reads would be detected as reads with “split mapping” signature and falsely identified as SGRs. The second and third criteria were therefore used to determine which of these singleton reads were unlikely to be artificially formed chimeras and prioritize them for subsequent experimental confirmation. Since chimeric reads can be formed by concatenation of two DNA fragments randomly sampled from the genome, the probability of finding two randomly sampled DNA fragments in a chimeric read should be independent of how distant the fragments are from each other in the genome. However, when we regarded all the deletion-or-duplication rearrangements as putative deletions and examined those with sizes less than 50 kb, small deletions (less than 5 kb) were significantly over-represented in the three datasets (Figure 3), which was the basis for the second criterion and the split distance threshold (5 kb) used to classify deletion-or-duplication rearrangements. For those deletion-or-duplication rearrangements with putative deletion sizes extending 5 kb, more than 95% were large deletions (>200 kb) (Table S1), which supported the classification of deletion-or-duplication rearrangements with a split distance more than 5 kb as duplications because a single deletion event as large as more than 200 kb has rarely been observed and is likely to be lethal [12]. The third criterion was based on the examination of the overlapping microhomologies at the putative rearrangement junctions and was used to select those putative rearrangements with junction microhomologies longer than expected for chimeric reads. The junction microhomology distribution analysis was performed on those singleton reads that fail to meet the first two criteria. The distributions of junction microhomology for the three datasets (gen48, gen144 and gen240) were heavily skewed towards the upper side compared to the simulated dataset (20 million in silico chimeric reads), which indicated that those rearrangement junctions with longer microhomologies were not due to artificially formed chimeras (Figure 4). As described in Materials and Methods, the cut-off junction microhomology for the third criterion was calculated to be ≥7 bp, ≥9 bp, and ≥8 bp for the three datasets gen48, gen144 and gen240 respectively. After screening using these three criteria, 104 putative rearrangements in gen48, 67 in gen144 and 25 in gen240 were selected as potential SGRs and a subset of those were further examined to confirm presence of the rearrangements (Figure 2C). In addition, we have also been able to identify the junction sequences led by the two site-specific inversions responsible for flagellar phase variation of *S. typhimurium* [13]. But these inversion junctions were not included in this study since their formation was mediated by site-specific recombinase.

### Experimental verification of putative rearrangement junctions

**Figure 5:**
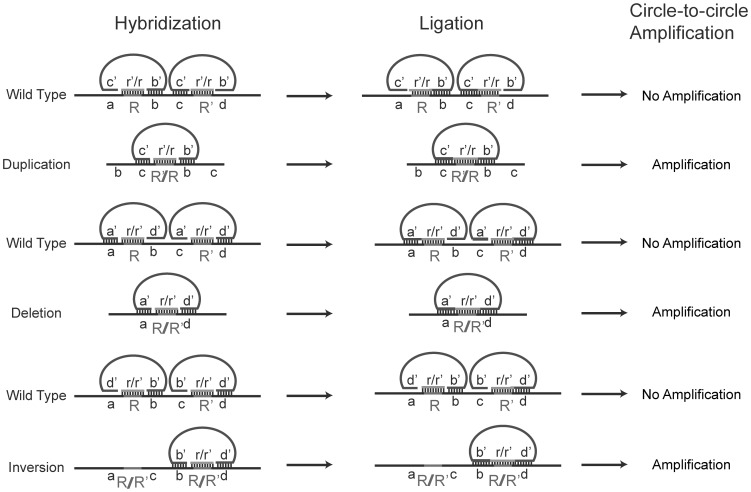
Design rationale for padlock probes. The two end segments of the padlock probes and the connector sequence were designed to be complementary to three consecutive sequences in the target rearrangement junction sequence. If the two end segments and connector sequences are perfectly hybridized a closed circular molecule can be formed by two ligations. For the wild type sequence, only one ligation can occur leading to a non-circularized molecule.

Due to the limitation of its sensitivity and validity, PCR was only used as an auxiliary confirmation method. A more sensitive probing technique was employed in this work to examine the existence of the putative SGRs. This detection approach was based on padlock probes, which are designed for circularization when bound to the correct target DNA sequences [14]. Coupled with rolling circle amplification (RCA) [15], padlock probing has been successfully used to detect single DNA molecules [16]–[18]. Since the putative rearrangements had short microhomologies at their junctions, the padlock probes were designed to have three segments complementary to target DNA sequences at rearrangement junctions. This allowed unambiguous detection of the low abundance junction sequences from the pool of wild type sequences (Figure 5). The formed circularized DNA was then used as templates for RCA (Figure S2). Based on the results from a pilot detection experiment by mixing known junction sequences with wild type sequence in different ratios (Figure S3A), this technique allowed us to detect rearrangements with a frequency as low as 0.001%. The detection sensitivity varied between different targeted junction sequences and even greater sensitivity could be achieved for certain junction sequences. Previous data suggest that fewer than 10 DNA molecules can be detected by padlock probe technology [19]. For those rearrangements with long junction microhomologies (>30 bp), padlock probes are not applicable due to the limited length of the probes. Using padlock probes and/or PCR, subsets of duplications (8/28), inversions (7/27) and putative deletions (15/49) from dataset gen48 were examined to confirm the presence of unique junction sequences (Table 1 and Table S1). The DNA used for these tests was the same DNA preparation as that used for whole-genome sequencing and the initial identification of the junction sequences. One difficulty encountered in experimental verification of rearrangement junction sequences led by SGRs is to find a proper negative control, which is genomic DNA prepared from the same bacterial cells but that does not contain the SGRs under investigation. As SGRs are spontaneously formed during bacterial growth this could possibly occur in any independent bacterial culture. Therefore, instead of using *S. typhimurium* genomic DNA, *E. coli* genomic DNA was used as the negative control for all the padlock probe detection experiments in this work given the frequent nucleotide differences between the two genomes. Nevertheless, one would expect SGRs to arise at very different frequencies in independent cultures in which SGRs have not been allowed to approach steady state frequencies. To test this, we randomly picked seven SGRs that were positively verified by padlock probes and performed the parallel detection experiments by using *S. typhimurium* genomic DNA prepared from two independent cultures that were grown for less than 10 generations (two overnight cultures from single colony inoculation), three SGRs were not detectable in at least one of the tested genomic DNA preps and the other four SGRs were detected in both DNA samples but the fluorescence signals were substantially different from the signal given by genomic DNA prepared from the chemostat culture gen48 (data not shown). This result indicated that the positive signals observed in padlock probe detection experiments were due to the existence of the true rearrangement junction sequences rather than an artifact generated by any genomic DNA irrespective of its origin. The number of putative SGRs successfully verified by padlock and/or PCR were: 5/8 tested duplications, 5/7 inversions and 12/15 deletions (Table 1 and Figure S3B–D). The deduced junction microhomology cut-off values based on the verification result were ≥11 bp, ≥12 bp and ≥8 bp for duplications, inversions and deletions respectively, which suggested that there were 12 duplications, 11 inversions and 28 deletions expected to be true genome rearrangements in the dataset gen48 (Figure 2D).

**Table 1:** Experimental verification of putative rearrangement junctions.

	Read Name	Junction microhomology	PCR&Sequencing	Padlock probe
Duplications	GFLSN1V01EIE4D	7 bp	ND*	**−**
	GFLSN1V02JDL3E	9 bp	ND	**−**
	GFLSN1V01CBH8U	11 bp	ND	**+**
	GFLSN1V02IGMQT	13 bp	**−**	**+**
	GFLSN1V02IPGBJ	14 bp	**−**	**+**
	GFLSN1V02FS66Y	16 bp	**−**	**+**
	GFLSN1V02JRTIW	17 bp	**−**	**−**
	GFLSN1V02IASJO	27 bp	**−**	**+**
Inversions	GFLSN1V01CX2KF	7 bp	ND	**−**
	GFLSN1V01C0BRM	9 bp	ND	**−**
	GFLSN1V02ITBVM	12 bp	ND	**+**
	GFLSN1V02HOFOR	13 bp	**−**	**+**
	GFLSN1V02HR39L	14 bp	**−**	**+**
	GFLSN1V02J57RN	15 bp	**−**	**+**
	GFLSN1V02GSR9L	18 bp	**−**	**+**
Deletions	GFLSN1V01DBBRA	1 bp	**−**	**−**
	GFLSN1V02F72TK	3 bp	ND	**−**
	GFLSN1V01BH8B1	5 bp	ND	**−**
	GFLSN1V01CDFB9	8 bp	ND	**+**
	GFLSN1V01DOBJK	12 bp	**+**	**+**
	GFLSN1V01BZFPJ	15 bp	ND	**+**
	GFLSN1V02HCA7P	17 bp	ND	**+**
	GFLSN1V01AK6D5	19 bp	ND	**+**
	GFLSN1V01E0QZI	28 bp	**+**	**+**
	GFLSN1V01DJFOY	32 bp	**+**	**+**
	GFLSN1V01A7KE5	44 bp	**+**	ND
	GFLSN1V02JG15X	78 bp	**+**	ND
	GFLSN1V01B9VTY	83 bp	**+**	ND
	GFLSN1V02JJGI4	102 bp	**+**	ND
	GFLSN1V01C22ZK	178 bp	**+**	ND

* ND: not determined.

### SGRs are very common and their frequencies rapidly approach steady state

**Figure 6:**
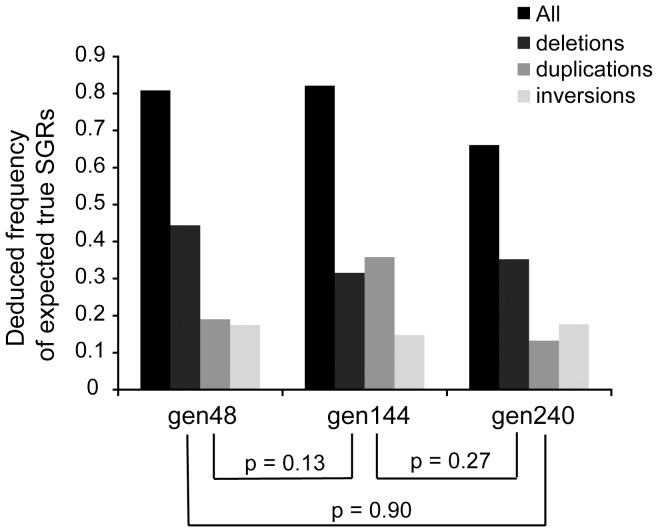
Deduced frequencies of expected true SGRs. The frequencies were calculated as the number of expected true SGRs divided by the sequencing coverage for the three datasets gen48, gen144 and gen240, respectively. The distributions of the three rearrangement events (deletion, duplication and inversion) were compared between each pair of the three datasets using chi-square two-sample test.

The frequency of SGRs was calculated as the number of expected true rearrangements (based on the verification results obtained from padlock probes and/or PCR) divided by sequencing coverage for the three datasets gen48, gen144 and gen240 (Figure 6). The distributions of the three rearrangement events for each pair of the three datasets were compared using chi-square two-sample test and there was no significant difference between any two of the three datasets (Figure 6). Given that the frequencies of all the rearrangement events are roughly the same for the three datasets, this indicated that the frequency of SGRs in the bacterial population had reached steady state already within 48 generations. This result is consistent with the observation in a recent genetic study, in which duplications reached the steady state frequency within about 30 generations of growth [20]. The frequencies of expected true duplications and inversions at generation 48 are both around 20% (Figure 6) and the true frequencies could be even higher given that some rearrangement junctions might be left undetected due to the detection limit of the padlock probe technique and limitations of the split mapping method in detecting rearrangement junctions with long microhomologies (e.g. spontaneous tandem duplications between rRNA operons). The deduced frequency of expected true small deletions at generation 48 was about 40% (Figure 6).

### Some genes included in identified deletions were found deleted when comparing different Salmonella serovars/subspecies

If the frequencies of small deletions were as high as suggested from our experimental results, one would expect that these deletions should also often be deleted when comparing different Salmonella genomes. To examine this idea, we compared the genomic sequence of *S. typhimurium* with 14 other closely related Salmonella serovars/subspecies (see Materials and Methods for list of strains). Among 4620 genes annotated in *Salmonella typhimurium* genome, 1213 genes were found completely or partially deleted in at least two other Salmonella serovars/subspecies (Table S2). In total 49 genes were included in the 43 identified small deletions that were either experimentally verified or expected to be true based on the verification result and 35/49 genes were found deleted in at least two other Salmonella serovars/subspecies, which is significantly higher than expected (Fisher's Exact Test, p = 7×10^−11^) (Table S3). These findings are compatible with our experimental data and detection of unique junctions and suggest that certain genes are highly prone to loss.

### Sequence analysis at rearrangement junctions

The sequences at 100 bp on either side of the 190 unique breakpoints obtained from the rearrangement junctions that were either experimentally verified or expected to be true based on the verification result in three datasets (Table S4), were examined for GC content and nucleotide tracts (polypyrimidines, polypurines and alternating purine-pyrimidine) and no remarkable signatures were observed. Similarly, none of the rearrangement junctions were located near any of the six IS*200* elements present in *Salmonella typhimurium* LT2. Palindromes are not expected true deletions (out of 86 unique breakpoints) 16 were located within intergenic regions. Given a coding sequence density of almost 90% in the *Salmonella typhimurium* LT2 genome, the deletions appeared enriched in intergenic regions (p = 0.01). This indicated that intragenic deletions might in general cause more severe fitness reductions and therefore counter-selected during growth and present at lower steady-state frequencies.

## Discussion

In this study, we show the utility of the 454 pyrosequencing technology and a ‘split mapping’ computational method to investigate SGRs in bacterial populations. Massively parallel pair-end sequencing has been extensively used to identify genome rearrangements in cancer genomes, in which putative rearrangements were suggested by discordantly mapping reads and then experimentally confirmed by PCR amplification of the breakpoints in tumor and normal DNA. However, the frequencies of non-selected SGRs in bacterial populations are usually very low, which renders PCR unreliable in verifying putative SGRs. Therefore, we used 454 pyrosequencing to obtain whole genome sequences in relatively long reads (300 nucleotides on average), subsequently determined the breakpoints of the putative SGRs to base pair resolution via a ‘split mapping’ computational method and employed a new technique, padlock probe hybridization, to experimentally verify the junction sequences of putative SGRs. By using this strategy, we were able to identify and experimentally confirm junction sequences caused by SGRs in a *S. typhimurium* population and determine how fast SGRs approach their steady state frequency by examining the frequency of SGRs at three different time points (generations 48, 144 and 240) in cells from a chemostat-grown population. We classified the identified putative rearrangements into duplications, inversions and small deletions based on the relative chromosomal locations and orientations of the prefix and suffix in a read sampled across a putative rearrangement. One should note that the junctions caused by translocations would also be identified as duplication or inversion junctions, but since they are rare rearrangement events in *S. typhimurium* it is likely that translocations only had a small contribution in generating junction sequences in the chromosome [21].

Based on the verification results, the frequency of expected true SGRs at generation 48 was calculated to be approximately 20%, 20% and 40% for duplications, inversions and small deletions, respectively (as estimated from dataset gen48) and SGRs reached steady state within 48 generations based on the observation that there was no significant difference between the three datasets (gen48, gen144 and gen240) in terms of the frequency of expected true SGRs. Previous estimates suggest that at least 10% of cells contain a duplication somewhere in the genome in a growing *S. typhimurium* culture [22]. The frequency of spontaneous duplications (20%) deduced from this study is in good agreement with the previous estimate (10%), considering that the previous calculation of duplication frequency was based on a subset of spontaneous duplications that could potentially bias the estimation. Our results suggest that spontaneous duplications are more frequent than previously estimated, even though the most frequent rearrangements (spontaneous duplications between rRNA operons) are not detectable in this study. Thus, the frequency of duplications is likely to exceed 20% of the cells in the population. To our knowledge, neither inversion or deletion frequency has been measured previously on a genome-wide scale in a bacterial genome because the detection usually relies on observable phenotypes generated by these two types of rearrangements and it is difficult to do on a large scale in the chromosome. Most previous works on measuring inversion frequencies were based on placing sequences in inverse order at known chromosomal positions and examining inversions formed at these specific sequences [23]–[26]. Furthermore, for measurements of deletion frequencies most studies were either performed in the same manner as inversions by placing sequences in direct order at known positions [27]–[30], or focused on deletions occurring within a specific sequence context [31]–[33]. Thus, the results of our study provide new insights into frequencies of SGRs in bacteria populations.

Despite the strength of this new strategy in terms of detecting and validating low abundance SGRs on a genome-wide scale in bacterial populations, a few limitations should be noted: (1) Because the frequencies of most SGRs are relatively low in a bacterial population, it is not possible to isolate individual cells with a particular genome rearrangement and study it in detail but instead we have to rely on identifying the unique junction sequences generated by the SGRs and deduce the structures of the rearrangements. (2) SGRs formed between long repetitive sequences are undetectable due to the limited read length, which could lead to underestimation of rearrangement frequencies. (3) Although padlock probe hybridization technique has been used to detect low abundance DNA sequences with extraordinary sensitivity and precision, we cannot completely rule out the existence of artifacts giving rise to false positives. One possibility is that, in detecting a small deletion junction sequence, the wild type sequence of deleted region forms a hairpin loop structure that could potentially juxtapose the padlock probes binding to the flanking regions and lead to a substrate for DNA ligation. However, we were unable to find any strong palindromic sequences in the small deletions identified in this work, which made this possibility less likely. (4) Padlock probe hybridization technique is not applicable for those rearrangements with >30 bp junction microhomology due to the limited length of padlock probe. Therefore, verification of rearrangements with long junction microhomology can only rely on PCR. The deduced frequencies of inversions (20%) and deletions (40%) are higher than expected considering the irreversibility of deletions and the low reversibility of inversions. If all the identified inversion and deletion junction sequences came from genomes of viable cells and these cells could form colonies on plates with proper size, one would expect that 60% of randomly picked colonies should contain an inversion or a deletion somewhere in the genome assuming that these rearrangements were evenly distributed among cells. However, the above deduction is unlikely to be true because otherwise it should have been noticed in whole genome re-sequencing work. This discrepancy can be explained by the fact that the examination of SGRs in this work was based on the detection of rearrangement junction sequences rather than isolation of mutants with selectable phenotypes as in most previous works on this subject. Firstly, cells with inversions or deletions, which are likely to be costly for the cells to carry [34], could be either very slow-growing or lethal and cannot form full-size colonies. Secondly, irreversible rearrangements could be accumulated in a small subpopulation of cells that each contains many different rearrangements, which will lead to two possible outcomes: (i) cells with multiple rearrangements cannot form full-size colonies due to the synthetic sickness or lethality; (ii) the small size of subpopulations with rearrangements will make it difficult for small-scale whole genome re-sequencing work to find those clones derived from cells with rearrangements, e.g whole-genome re-sequencing of 100 independent clones each derived from a single cell is required to detect such a clone if 1% of cells in the population accumulate rearrangements.

In a summary, by using the strategy described in this work, we have taken three “snapshots” of a growing bacterial population at three transient states (generations 48, 144 and 240) in terms of their genomic sequences and revealed all the footprints (junction sequences) made by chromosomal rearrangements at each of the transient states, but these footprints might disappear under certain conditions, such as forming visible colonies on plates. Finally, we think that complete characterization of all types of SGRs in unselected bacterial populations will require combining pair-end sequencing of libraries with large insert size (10 kb), which can be used to detect rearrangements between large repeats (rRNA operons, IS elements), and the strategy described in this study. With the fast development of sequencing technologies, one would expect more rapid and accurate estimate of the frequency of SGRs in bacterial populations under any defined genetic background or growth conditions, which could greatly facilitate examination of genome stability and studies of bacterial genome evolution.

## Materials and Methods

### Bacterial growth and sample collection

The bacterial strain used in this study was *Salmonella enterica* Var. Typhimurium LT2. EZ Rich Defined Medium Kit (M2105, TEKNOVA) was the growth medium. A 20 ml overnight culture was initiated from <10 cells of *S. typhimurium* and transferred to a chemostat. The doubling time was set to 30 minutes by adjusting the dilution rate and the chemostat culture was grown for 240 generations. Fifteen ml chemostat cultures were collected at generation 48, 96, 144, 192, 240. One ml culture was stored at −80°C and the rest were used for preparation of genomic DNA.

### 454 pyrosequencing

Genomic DNA was prepared for sample gen48, gen144 and gen240 using Genomic-tip 500/G (QIAGEN) according to the manufacture's instructions. Genome sequencing was performed with a Genome Sequencer FLX (Roche) at the KTH Sequencing Facility, Royal Institute of Technology, KTH, Stockholm, Sweden. The raw sequencing data were deposited in NCBI Sequence Read Archive (http://www.ncbi.nlm.nih.gov/Traces/sra/) and the accession numbers are SRX156388, SRX156390, and SRX156391 for the three datasets gen48, gen144 and gen240 respectively.

### Identification of junctions by split mapping

A local database was created for *S. tyhimurium* LT2 genomic sequence using FORMATDB (ftp://ftp.ncbi.nih.gov/blast/) and all sequencing reads were blasted against this local database using BLASTALL (ftp://ftp.ncbi.nih.gov/blast/). A read spanning a rearrangement junction will leave a ‘split mapping’ signature in the reference genome, with a prefix and suffix of the read mapped to different genomic locations. Reads with such a signature were mined from the blast result using custom Perl scripts (Scripts S1). The two perfect matches (with the lowest E value) corresponding to the prefix and suffix were used to infer the orientations and relative chromosomal locations of the two split fragments. A read was set aside if there was more than one perfect match for either the prefix or suffix of the read. Overlapping microhomologies at rearrangement junctions were determined for each putative spontaneous genomic rearrangement based on the relative positions of the two perfect matches in the reads.

### Artifacts removal and quality analysis

Reads that were mapped to identical genomic locations were considered as PCR duplicates created during PCR enrichment step and only the one with highest quality score was retained. Both the five bases on either side of a junction (excluding overlapping region) and the overlapping region (only for those with ≥5 bp junction microhomology) were required to have an average Phred score of 20 or higher, unless support for a putative rearrangement was indicated by additional reads, and only these were selected for further confirmatory screening.

### Confirmatory screening

The following criteria were used to prioritize putative rearrangements for confirmatory PCR and padlock probes detection: (i) ≥2 reads spanning the same rearrangement; (ii) reads designating small deletions (<5 kb) based on the skewed size distribution of putative deletions; (iii) reads with microhomologies at the junctions significantly longer than expected. The third criterion was used to exclude those chimeric reads possibly formed by direct ligation of two DNA fragments during the library construction. The procedures are as follows: the putative rearrangements (excluding those meeting the first two criteria) were categorized into thirteen groups based on the length of overlapping microhomologies at the junctions (0–11 bp, and >11 bp) and the proportion of each category was calculated; a chimeric read formed by direct ligation of two DNA fragments can be mimicked by concatenating two randomly picked 200 bases from both plus and minus strand of the genomic sequence. The probability of a chimeric read having the junction microhomology in each of the thirteen categories (0–11 bp, and >11 bp) was calculated by accumulative sampling and analysis of up to 20 million in silico chimeric reads, which is used to calculate the expected number of reads falling in each junction microhomology category by multiplying the total number of reads spanning a putative rearrangement junction. The 95% confidence interval of the number of putative rearrangements falling in each of the thirteen junction microhomology category was calculated based on the observed value using binomial distribution. The cut-off was set to be the junction microhomology (bp) where the minimum value in the 95% confidence interval was ≥10 fold larger than the expected number of reads falling in each junction microhomology category.

### Padlock Probes Hybridization

All oligonucleotides used in the padlock probe assay were ordered from IDT (sequences listed in Table S5). Prior to the probing assays, all padlock probes and connector oligonucleotides were phosphorylated. Briefly, 100 µl of phosphorylation mixture containing 1 µM of padlock probes, 1× PNK buffer A (Fermentas), 1 mM ATP (Fermentas), 0.1 U/µl T4 Polynucleotide Kinase (Fermentas) was incubated at 37°C for 30 min and 65°C for 20 min using a thermo cycler. These probes can be stored at −20°C until used. Before mixing with the ligation mixture, 5 µl samples containing a total amount of 200 ng purified bacteria DNA were first incubated at 95°C for 5 min, and immediately chilled on ice. This is followed by adding of 5 µl ligation mix containing 1×Ampligase buffer (Epicentre), 2.5 U Ampligase (Epicentre), 100 pM of corresponding padlock probe and 100 pM of connector oligonucleotides. The ligation was carried out at 55°C overnight, forming DNA circles. The resulting DNA circles were thereafter amplified by the first generation RCA by adding 5 µl RCA mix containing 1×phi29 DNA polymerase buffer (Fermentas; 33 mM Tris-acetate (pH 7.9 at 37°C), 10 mM Mg-acetate, 66 mM K-acetate, 0.1% (v/v) Tween-20, 1 mM DTT), 100 µM dNTPs, 0.2 mg/ml BSA and 2 U phi29 DNA polymerase. The mix was incubated at 37°C for 1 hour followed by 1 min at 65°C to inactivate the phi29 DNA polymerase. This was followed by monomerization of the amplified single molecules. Five µl restriction digestion mixture containing 1 U/µl *Alu*I restriction enzyme (NEB), 1×phi29 DNA polymerase buffer, 400 nM replication oligonucleotides RO+, and 0.2 mg/ml BSA. The reaction was carried at 37°C for 1 min and *Alu*I was inactivated at 65°C for 1 min. The monomers were then recircularized and amplified to generate second generation RCA products by adding 5 µl ligation and RCA mix containing 1× phi29 DNA polymerase buffer, 0.2 mg/ml BSA, 2 mM ATP, 0.25 mM dNTP, 0.1 U/µl T4 DNA ligase (Fermentas) and 2 U phi29 DNA polymerase. Then, 5 µl restriction digestion mix containing 1 U/µl units *AluI*, 1× phi29 DNA polymerase buffer, 1.6 µM replication oligonucleotides RO-, 0.2 mg/ml BSA was added. Finally, the third RCA were then initiated by adding the ligation and RCA mix again.

After three generation of RCAs, 65 µl detection mix was added into the RCA products, resulting in final concentrations: 20 mM Tris-HCl (pH 8.0), 20 mM EDTA (pH 8.0), 1 M NaCl, 0.1% Tween-20 and 5 nM detection probes. The hybridization was carried out at 80°C for 1 min and 65°C for 10 min.

After hybridization with detection probes, the RCA products can be visualized as individual fluorescent dots by using the confocal microscopy, these dots can therefore be quantified in a digital approach. The detection process was accomplished by a microfludic based digital quantification system as described by Jarvius, *et al* [19].

### PCR and sequencing

Primers were designed to span the possible breakpoints. PCR reactions were performed on 100 ng genomic DNA used for 454 genome sequencing. Products giving a band were sequenced by conventional Sanger capillary methods (Eurofins MWG/Operon) and compared to the reference genome to identify the breakpoints (sequences listed in Table S6).

### Genome comparisons

Genomic sequences of fourteen *Salmonella*, including thirteen enterica serovars (*Enteritidis*[GenBank: AM933172.1], *Typhi* [GenBank: AL513382.1], *Schwarzengrund* [GenBank: CP001127.1], *ParatyphiA* [GenBank: CP000026.1], *ParatyphiB* [GenBank: CP000886.1], *ParatyphiC* [GenBank: CP000857.1], *Heidelberg* [GenBank: CP001120.1], *Gallinarum* [GenBank: AM933173.1], *Dublin* [GenBank: CP001144.1], *Choleraesuis* [GenBank: AE017220.1], *Arizonae* [GenBank: CP000880.1], *Agona* [GenBank: CP001138.1], and *Newport* [GenBank: CP001113.1]) and one subspecies (*Bongori* [GenBank: FR877557.1]) were compared to *S. typhimurium* [GenBank: AE006468.1]. The fourteen pairwise comparisons were performed using Mauve (http://gel.ahabs.wisc.edu/mauve/). Each comparison generated a backbone file, which was used to infer the potential deletions in these fourteen strains compared to *S. typhimurium*. The complete list of deleted genes and the number of genomes in which each specific gene was found deleted was compiled in Table S2.

### Sequence analysis at junctions

In total 869 unique breakpoints extracted from all putative rearrangements (meeting the first two confirmatory criteria) were used in the analysis of breakpoint sequence context, excluding overlapping regions and insertions. 10 bp and 100 bp of genomic sequences on either side of the breakpoint sites were compared to 100 sequences of the same length sampled from a 20 kb region surrounding the breakpoint but excluding the breakpoint sequence itself. Differences in the length of nucleotide tracts (polypurine/polypyrimidine and alternating purine/pyrimidine) were tested using Mann-Whitney U-test and the average GC content was compared using Fisher exact test.

## Supporting Information

Figure S1 **Illustration of split mapping and classification for putative rearrangements.** The split read has the prefix and suffix mapped to different locations on the reference genome. The prefix and suffix are defined as the first and second split segments coming in the read and have no indication of the mapping orientations. The basic signatures include (i) inversion, where the two split segments are mapped in different orientations, (ii) deletion or duplication, where the two split fragment are mapped in the same orientation. A small split distance (from the prefix to the suffix along the mapping orientation) makes deletion-or-duplication rearrangements more likely to be deletions and a large split distance makes such rearrangements more likely to be duplications.
(TIF)

Figure S2 **Principle of rolling circle amplification (RCA).** 1a) Padlock probes and connector oligonucleotides were added to samples and hybridized to the correct template. 1b) Padlock probes and connector oligonucleotides were then ligated by DNA ligase to form a completed DNA circle. 2) Ligated padlock probes were amplified by RCA. 3a) At the presence of restriction oligonucleotides, RCA products were digested by restriction enzyme to generate monomers. 3b) The monomers hybridize head-to-tail with the excess amount of restriction oligonucleotides. 4) The monomers become circularized through DNA ligation. 5) New DNA circles are amplified with RCA to generate 2nd generation of RCA products. 6) Second digestion of RCA products to generate monomers again. 7) Monomers were re-circularized and again amplified by RCA to generate third generation RCA products. 8) The third generation RCA products were hybridized to fluorescence labeled detection oligonucleotides. The fluorescence labeled detection oligonucleotides RCA products can be detected in a digital quantification system.
(TIF)

Figure S3 **Padlock probe detection of rearrangement junctions.** (A) Genomic DNA from each of four deletion mutants (Del1, Del2, Del3 and Del4) was mixed with wild type *S. typhimurium* genomic DNA in three different mutant/wt ratios: 1%, 0.1% and 0.001%. Padlock probes were designed according to the endpoints of the deletions (Table S5) and the detection experiment was performed on both wild type DNA and mixture of mutant and wild type DNA. (B, C, and D) For each padlock probe, the detection experiment was performed on both *S. typhimurium* (abbreviated as Sty in the figure) genomic DNA (used for 454 pyrosequencing) and *E. coli* (abbreviated as Eco in the figure) genomic DNA as negative control. The detection was regarded as positive if the fluorescence counts was more than 1000 and significantly higher than negative control.
(TIF)

Table S1 **List of putative rearrangements with ‘split mapping’ signature.**
(XLSX)

Table S2 **List of genes that were found deleted in genome comparison analysis between** ***S. typhimurium*** **and other Salmonella subspecies/serovars.**
(XLSX)

Table S3 **List of genes included in identified deletions and the times being found deleted in genome comparison study between S. typhimirum and other Salmonella subspecies/serovars.**
(XLS)

Table S4 **List of expected true rearrangements based on experimental verification result and their breakpoints.**
(XLSX)

Table S5 **Oligonucleotides for padlock probe assays.**
(DOCX)

Table S6 **Oligonucleotides for PCR amplification and Sanger sequencing.**
(DOCX)

Scripts S1 **Custom perl scripts used split-read mapping filtering, sequencing quality analysis, and generation of in silico chimeric reads.**
(ZIP)
