# Supplementary figures and images for: Genome-Wide Detection of Spontaneous Chromosomal Rearrangements in Bacteria
Source: PLoS One. 2012 Aug 3;7(8):e42639. doi: 10.1371/journal.pone.0042639 (PMC3411829; doi:10.1371/journal.pone.0042639)

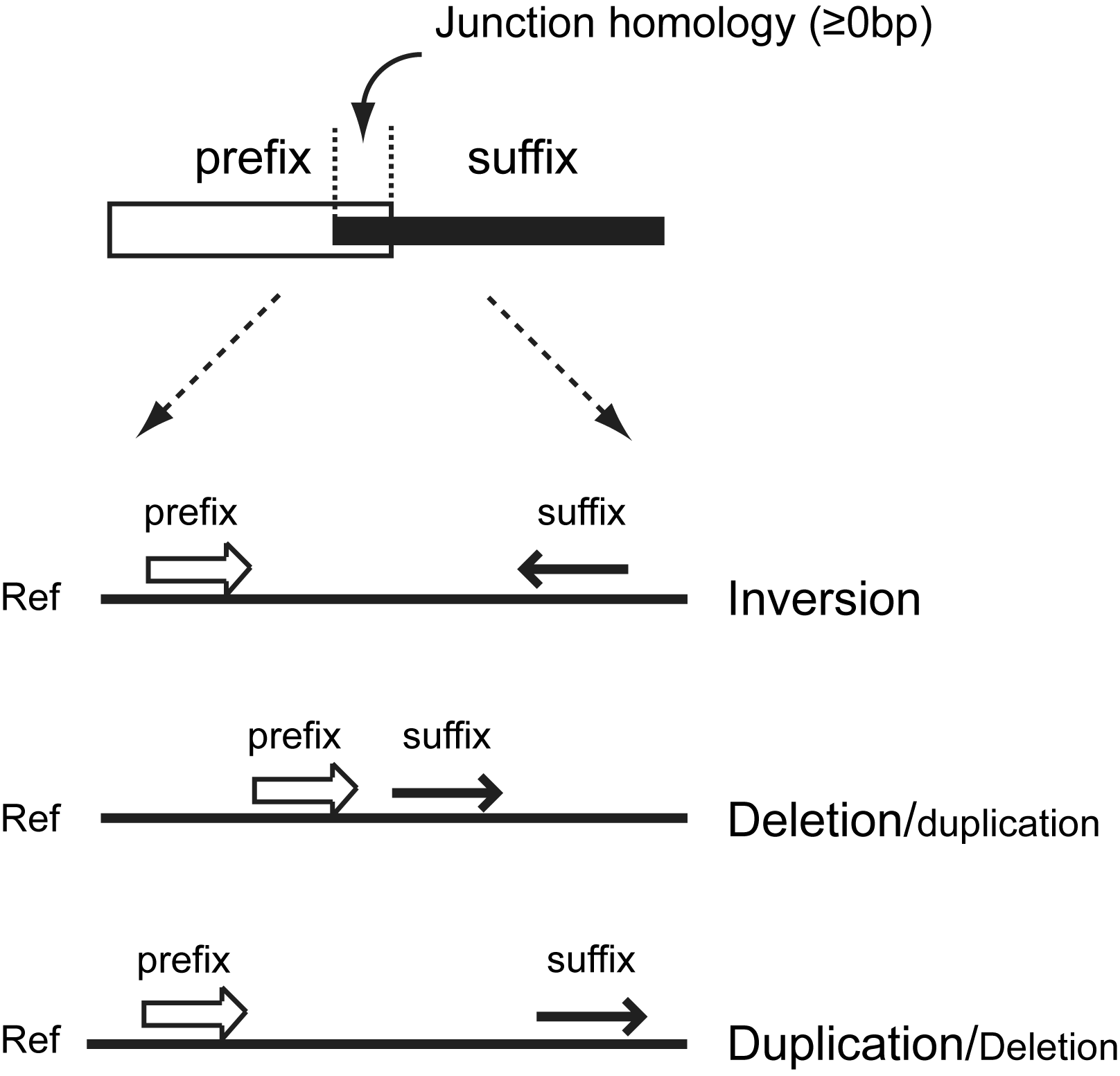

Supplement: Figure S1 — Illustration of split mapping and classification for putative rearrangements. The split read has the prefix and suffix mapped to different locations on the reference genome. The prefix and suffix are defined as the first and second split segments coming in the read and have no indication of the mapping orientations. The basic signatures include (i) inversion, where the two split segments are mapped in different orientations, (ii) deletion or duplication, where the two split fragment are mapped in the same orientation. A small split distance (from the prefix to the suffix along the mapping orientation) makes deletion-or-duplication rearrangements more likely to be deletions and a large split distance makes such rearrangements more likely to be duplications. (TIF) [file pone.0042639.s001.tif]

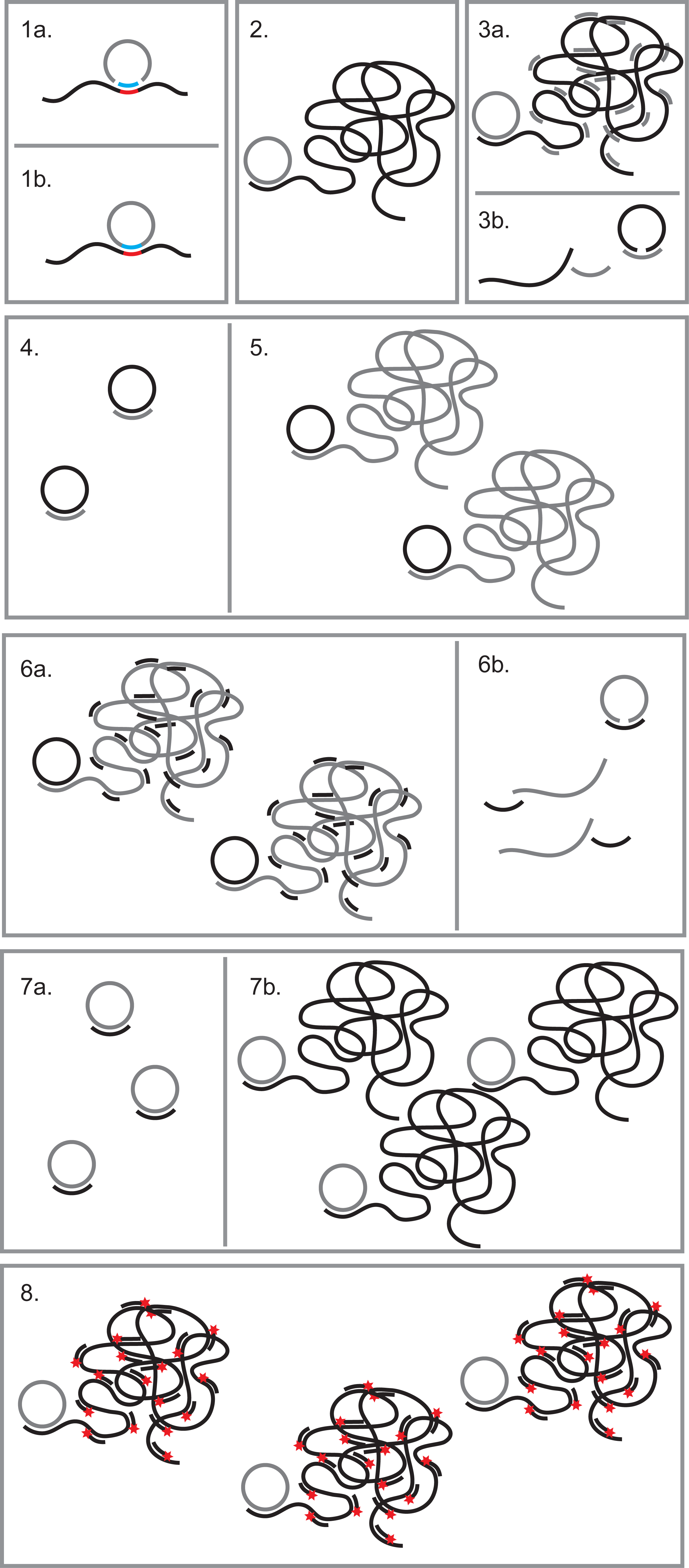

Supplement: Figure S2 — Principle of rolling circle amplification (RCA). 1a) Padlock probes and connector oligonucleotides were added to samples and hybridized to the correct template. 1b) Padlock probes and connector oligonucleotides were then ligated by DNA ligase to form a completed DNA circle. 2) Ligated padlock probes were amplified by RCA. 3a) At the presence of restriction oligonucleotides, RCA products were digested by restriction enzyme to generate monomers. 3b) The monomers hybridize head-to-tail with the excess amount of restriction oligonucleotides. 4) The monomers become circularized through DNA ligation. 5) New DNA circles are amplified with RCA to generate 2nd generation of RCA products. 6) Second digestion of RCA products to generate monomers again. 7) Monomers were re-circularized and again amplified by RCA to generate third generation RCA products. 8) The third generation RCA products were hybridized to fluorescence labeled detection oligonucleotides. The fluorescence labeled detection oligonucleotides RCA products can be detected in a digital quantification system. (TIF) [file pone.0042639.s002.tif]

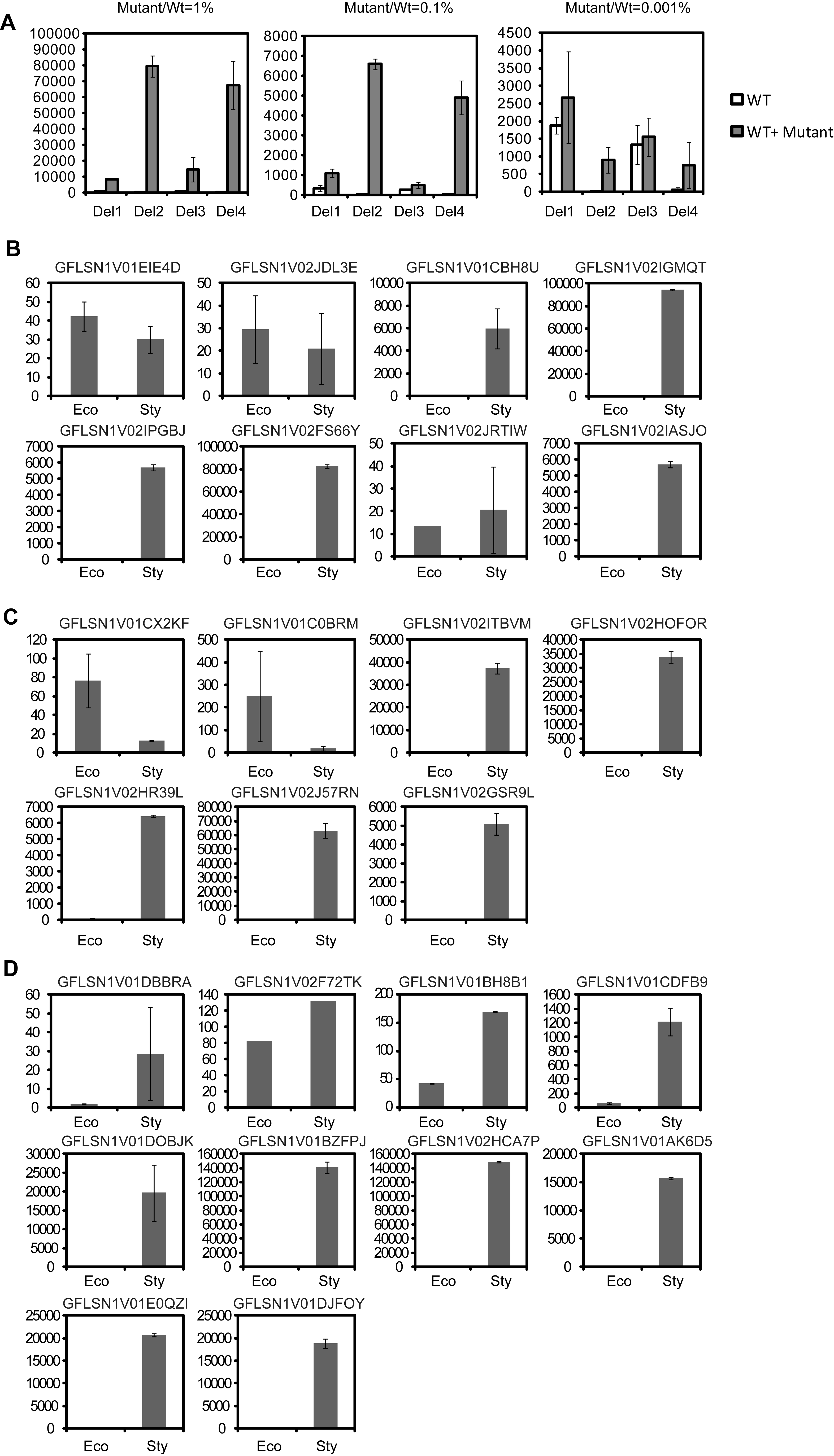

Supplement: Figure S3 — Padlock probe detection of rearrangement junctions. (A) Genomic DNA from each of four deletion mutants (Del1, Del2, Del3 and Del4) was mixed with wild type S. typhimurium genomic DNA in three different mutant/wt ratios: 1%, 0.1% and 0.001%. Padlock probes were designed according to the endpoints of the deletions (Table S5) and the detection experiment was performed on both wild type DNA and mixture of mutant and wild type DNA. (B, C, and D) For each padlock probe, the detection experiment was performed on both S. typhimurium (abbreviated as Sty in the figure) genomic DNA (used for 454 pyrosequencing) and E. coli (abbreviated as Eco in the figure) genomic DNA as negative control. The detection was regarded as positive if the fluorescence counts was more than 1000 and significantly higher than negative control. (TIF) [file pone.0042639.s003.tif]
