# Supplementary material for: Genome-Wide Detection of Spontaneous Chromosomal Rearrangements in Bacteria
Source: PLoS One. 2012 Aug 3;7(8):e42639. doi: 10.1371/journal.pone.0042639 (PMC3411829; doi:10.1371/journal.pone.0042639)
Supplement: Table S5 — Oligonucleotides for padlock probe assays. (DOCX) [file pone.0042639.s008.docx]

Table S5. Oligonucleotides for padlock probe assays.

| Type | Read Name | Sequence | Function |
| --- | --- | --- | --- |
| Four deletions used for spike-in experiments | Del1(330085-353239)*  Del2(2365072-2370134)*  Del3(2356258-2373236)*  Del4(2059793-2072105)*  Del1  Del3 | GCTGCTACCGCGGCTTTCACTGTCTATGTTTACAGCGGGCGTGTATGCAGCTCCTCAGTAGTGGATAGTGTCTTACACGATCTGACGTGTCAATGGCGGT GTTTTAACCATTCACGCAGTTGCGTCTATTTAGTGGAGCCGTGTATGCAGCTCCTCAGTAGTGGATAGTGTCTTACACGATCAACACATGCGCATCTAAT  ATGAAAATGTCTTCGCCAGCTGCGTCTATTTAGTGGAGCCGTGTATGCAGCTCCTCAGTAGTGGATAGTGTCTTACACGAACTCAGACTGTGCGGCGATG  CGCCGCGTAACCGCCATGGCCTTCGGGTTGTAAAGTAGTGTATGCAGCTCCTCAGTAGTGGATAGTGTCTTACACGACCCTGGTTAATGAAAGCATT  ACTGGGCGGCAGCCTGCGCC  GGCTCCGCCTGGCGCGCTGA | Padlock probe  Padlock probe  Padlock probe  Padlock probe  Connector oligonucleotides  Connector oligonucleotides |
| Duplication | GFLSN1V01EIE4D | CCATCACGATCACCGCCGTGGATAGTGTCTTACACGAAGAGTGTACCGACCTCAGTAAGTCTCCTAGCTCGGTGAACTGATTTCACGCAGCACGTC | Padlock probe |
|  | GFLSN1V02JDL3E | GCCTGCTCCAGCAGCTGTGGATAGTGTCTTACACGAAGAGTGTACCGACCTCAGTAAGTCTCCTAGCTCGGTGAACTTTTTTGACGGGAATCGCCT | Padlock probe |
|  | GFLSN1V01CBH8U | TCCACCACATGGTAGGCTTTTGCGTCTATTTAGTGGAGCCGTGTATGCAGCTCCTCAGTAGTGGATAGTGTCTTACACGAACTGCGCGTTTCTCCACCTC | Padlock probe |
|  | GFLSN1V02IGMQT | TCATCTCGGATATCGTCTTTTGCGTCTATTTAGTGGAGCCGTGTATGCAGCTCCTCAGTAGTGGATAGTGTCTTACACGAGCGATGCCGATACCCATCGA | Padlock probe |
|  | GFLSN1V02IPGBJ | GTATCGCCAACCTTTGTACTTGCGTCTATTTAGTGGAGCCGTGTATGCAGCTCCTCAGTAGTGGATAGTGTCTTACACGAAAATTACGGACTATATTTCC | Padlock probe |
|  | GFLSN1V02FS66Y | CTAACCGCCGTTATTCGTAATGCGTCTATTTAGTGGAGCCGTGTATGCAGCTCCTCAGTAGTGGATAGTGTCTTACACGAATATGGGTGTCGGTTGCCTG | Padlock probe |
|  | GFLSN1V02JRTIW | GTAGCGTCTTATCCGGCCTATGCGTCTATTTAGTGGAGCCGTGTATGCAGCTCCTCAGTAGTGGATAGTGTCTTACACGAATCCGGCATTGGCGCCACAT | Padlock probe |
|  | GFLSN1V02IASJO | CGTGCAATAGCATCCACGCATGCGTCTATTTAGTGGAGCCGTGTATGCAGCTCCTCAGTAGTGGATAGTGTCTTACACGAAAGCGAGAAAGGCGTTGCAG | Padlock probe |
|  | GFLSN1V01EIE4D | CAGATACGCGCCGACAATCG | Connector oligonucleotides |
|  | GFLSN1V02JDL3E | CAATGGCCAGACCGTACACC | Connector oligonucleotides |
|  | GFLSN1V01CBH8U | AGACGCCAGTTCGGCTTCA | Connector oligonucleotides |
|  | GFLSN1V02IGMQT | CATGCCGTGATAACGCTGC | Connector oligonucleotides |
|  | GFLSN1V02IPGBJ | CGATCCGCTGTTTATTCTGT | Connector oligonucleotides |
|  | GFLSN1V02FS66Y | CTCCCTATGATATCGCGCCG | Connector oligonucleotides |
|  | GFLSN1V02JRTIW | GATTGCCGGATGACGACGCA | Connector oligonucleotides |
|  | GFLSN1V02IASJO | CAGTAACCGGTTTTCTTCGCGCAGCCG | Connector oligonucleotides |
| Inversion | GFLSN1V01CX2KF | GCGCCGCAGGTTCGCCTGATGTGGATAGTGTCTTACACGAAGAGTGTACCGACCTCAGTAAGTCTCCTAGCTCGGTGAACTACAGTTGCTGCGCGCACGCA | Padlock probe |
|  | GFLSN1V01C0BRM | CAGACGCTGACAAAACGCGTGGATAGTGTCTTACACGAAGAGTGTACCGACCTCAGTAAGTCTCCTAGCTCGGTGAACTCGATAATAACTGCGCCATCA | Padlock probe |
|  | GFLSN1V02ITBVM | TCCTCGGCCTGTCCGGCGTTTGTCTATGTTTACAGCGGGCGTGTATGCAGCTCCTCAGTAGTGGATAGTGTCTTACACGAGGACTGAGCCATCTGATTCA | Padlock probe |
|  | GFLSN1V02HOFOR | GTTCATCGCTGAACTGATGTTGTCTATGTTTACAGCGGGCGTGTATGCAGCTCCTCAGTAGTGGATAGTGTCTTACACGATGCTAAAACGCGGCGTTCAG | Padlock probe |
|  | GFLSN1V02HR39L | AGCCACGGCAACAACGAGGCTGTCTATGTTTACAGCGGGCGTGTATGCAGCTCCTCAGTAGTGGATAGTGTCTTACACGAAACTGGCTTAATATGACCCC | Padlock probe |
|  | GFLSN1V02J57RN | CGAAAGCGGAAAGGTATAAGTGTCTATGTTTACAGCGGGCGTGTATGCAGCTCCTCAGTAGTGGATAGTGTCTTACACGAGGCTATCTTTAAATAACTGA | Padlock probe |
|  | GFLSN1V02GSR9L | TACGATTACTGTGATTACGATGTCTATGTTTACAGCGGGCGTGTATGCAGCTCCTCAGTAGTGGATAGTGTCTTACACGATTAAAAGCCGGATAGCCTGA | Padlock probe |
|  | GFLSN1V01CX2KF | TCGGCGGCCAGTACGTGCTC | Connector oligonucleotides |
|  | GFLSN1V01C0BRM | AACGCTCGCGCGGCGAGTGG | Connector oligonucleotides |
|  | GFLSN1V02ITBVM | AATCTCCATTCCGGTGGAAA | Connector oligonucleotides |
|  | GFLSN1V02HOFOR | TATTGTCAGGGATGGCCCTC | Connector oligonucleotides |
|  | GFLSN1V02HR39L | CGCGCAACGCGCTGGCGGCA | Connector oligonucleotides |
|  | GFLSN1V02J57RN | AAGCGGCCATCGCCATTGT | Connector oligonucleotides |
|  | GFLSN1V02GSR9L | TAGCGCAGCGCCATCAGGCC | Connector oligonucleotides |
| Deletions | GFLSN1V01DBBRA  GFLSN1V02F72TK | CTGGTAGTGGAGGACTAAAGCAGCGAACCATACGTGACGTGTATGCAGCTCCTCAGTAGTGGATAGTGTCTTACACGACTATCTTCGGTCGTGCGA  CAGCGCTTCAGGCAACAGAGCAGCGAACCATACGTGACGTGTATGCAGCTCCTCAGTAGTGGATAGTGTCTTACACGAGAACCGCCGGGTGTTTAC | Padlock probe |
|  | GFLSN1V01BH8B1 | ACATGCGCCGCCATGGAAAGCAGCGAACCATACGTGACGTGTATGCAGCTCCTCAGTAGTGGATAGTGTCTTACACGAGCCCGATGCCACGGCAGA | Padlock probe |
|  | GFLSN1V01CDFB9 | CTGAATTAAATGCTCTTTAGCAGCGAACCATACGTGACGTGTATGCAGCTCCTCAGTAGTGGATAGTGTCTTACACGAAATGTCCCCATGGTTAAC | Padlock probe |
|  | GFLSN1V01DOBJK | CTGAAAAAAGAGTGGGCTAAAGCAGCGAACCATACGTGACGTGTATGCAGCTCCTCAGTAGTGGATAGTGTCTTACACGAATGTCACCAACGGCATTACA | Padlock probe |
|  | GFLSN1V01BZFPJ | TATTGCGGCGCGTGGTCTGGTGCGTCTATTTAGTGGAGCCGTGTATGCAGCTCCTCAGTAGTGGATAGTGTCTTACACGATGAAACTGCGCGGCGGCGTG | Padlock probe |
|  | GFLSN1V02HCA7P | ACAAACTGTGGTGTGGATACTGCGTCTATTTAGTGGAGCCGTGTATGCAGCTCCTCAGTAGTGGATAGTGTCTTACACGAGCACTGATGTATATGCTGGG | Padlock probe |
|  | GFLSN1V01AK6D5 | GGCACGGTGCCCGGCCATGCGTCTATTTAGTGGAGCCGTGTATGCAGCTCCTCAGTAGTGGATAGTGTCTTACACGAATCATCCTCCACCCTCACCG | Padlock probe |
|  | GFLSN1V01E0QZI | AACGTCCGGCGTTGATCCAGCAGCGAACCATACGTGACGTGTATGCAGCTCCTCAGTAGTGGATAGTGTCTTACACGACTGTGCTCTGATTTATGA | Padlock probe |
|  | GFLSN1V01DJFOY | CTTTACCAACAGTAAGGGGGTGCGTCTATTTAGTGGAGCCGTGTATGCAGCTCCTCAGTAGTGGATAGTGTCTTACACGACAGTCCGGACACGCCG | Padlock probe |
|  | GFLSN1V01CDFB9 | GCCAGATCGCCGCCGCCGAA | Connector oligonucleotides |
|  | GFLSN1V01DOBJK | CCGCGTCGCTGGATAAAACG | Connector oligonucleotides |
|  | GFLSN1V01BZFPJ | GATGTGCTGGTGGCGACGGA | Connector oligonucleotides |
|  | GFLSN1V02HCA7P | GGTGGAGGATGCGACAGAGA | Connector oligonucleotides |
|  | GFLSN1V01AK6D5 | TCAGCGTATAGTCGCCATCC | Connector oligonucleotides |
|  | GFLSN1V01E0QZI | CCCGGAAATGCTGATTCTTGATGAGCC | Connector oligonucleotides |
|  | GFLSN1V01DJFOY | AAGGCCAACTACTGGGGCCATATGCCGGAAAC | Connector oligonucleotides |
| Oligonucleotides for replication and detection | RO+ | GTGTATGCAGCTCCTCAGTA | Restriction  oligonucleotides |
|  | RO- | TACTGAGGAGCTGCATACAC | Restriction  oligonucleotides |
|  | DO | Cy3-GTGGATAGTGTCTTACACGA | Detection probe |

*The endpoints of the four deletions used for spike-in experiments are indicated in the parentheses.
