# Supplementary material for: Genome-Wide Detection of Spontaneous Chromosomal Rearrangements in Bacteria
Source: PLoS One. 2012 Aug 3;7(8):e42639. doi: 10.1371/journal.pone.0042639 (PMC3411829; doi:10.1371/journal.pone.0042639)
Supplement: Table S6 — Oligonucleotides for PCR amplification and Sanger sequencing. (DOCX) [file pone.0042639.s009.docx]

Table S6. Oligonucleotides for PCR amplification and Sanger sequencing.

|  | Read Name | Forward Primer | Reverse Primer |
| --- | --- | --- | --- |
| Duplications | GFLSN1V02IGMQT | GCGTAATGCCGATAGCGAT | TACACTCTGGGCGTGCTGAT |
|  | GFLSN1V02IPGBJ | GATCGCCTGCCACCCATA | TCGAGGCCGTATATGTCCAT |
|  | GFLSN1V02FS66Y | GACGGGTTTGATATGGGTGT | GCGTTCCATTCCCAGACTTA |
|  | GFLSN1V02JRTIW | GTTGGCGGCTGCACAATAT | GGGCAAAGCGCATCTGTAG |
|  | GFLSN1V02IASJO | ATCTTCGCAATGGCGCAT | CAGCTCAAACAACTTGGTGTG |
| Inversions | GFLSN1V02HOFOR | CCGGCTATTCGAACTTGCAT | AGGGTCATCGGCACAGAATC |
|  | GFLSN1V02HR39L | CTGTGGTCCGATCCGTTCCT | GAACTGGTCGAGGCGGTAA |
|  | GFLSN1V02J57RN | ACAACATCCAGCTCGCAATAC | GGTGGAGAAGTAATGGAACTGCT |
|  | GFLSN1V02GSR9L | CAGCGGTCTGCATAACTACGT | TAGAAGCCGAAAGCGTCTTC |
| Deletions | GFLSN1V01DBBRA | GCAGACTTTAACGGCGTTGT | CAGGGCAATAGCTTCGTTG |
|  | GFLSN1V01DOBJK | TCCCGGAATATCACCAGCTT | GATCAACTGGTCGAGATCGTTAG |
|  | GFLSN1V01E0QZI | GACATTCGGTCGCAACGTT | ACGTTATCGGTTTATGAGAACGT |
|  | GFLSN1V01DJFOY | AAATCATTTACCGTCGGCAA | GGCGGCGATAGAACACAA |
|  | GFLSN1V01A7KE5 | GACCACTGACGAACTGAATGAG | CTTTAAATCAGCCTGAACGGTA |
|  | GFLSN1V02JG15X | ATGCGTACCATGCGACCA | CATACGGTGGGACTACCTGAA |
|  | GFLSN1V01B9VTY | GTCCGAAGTAATGAAATGCGA | CTGTCGTTGCGGACCAGA |
|  | GFLSN1V02JJGI4 | GTGTCTCAGAACACCGCTGAT | GAACGCACCCTCTTCGTCAT |
|  | GFLSN1V01C22ZK | CGGTCACTGTTGATACCCAAA | CAGGTTATCGCCTTTTGTTCC |
