# Supplementary material for: Genome-Wide Detection of Spontaneous Chromosomal Rearrangements in Bacteria
Source: PLoS One. 2012 Aug 3;7(8):e42639. doi: 10.1371/journal.pone.0042639 (PMC3411829; doi:10.1371/journal.pone.0042639)
Supplement: Scripts S1 — Custom perl scripts used split-read mapping filtering, sequencing quality analysis, and generation of in silico chimeric reads. (ZIP) [file pone.0042639.s010.zip › FORMATDB.rtf]

I am just going to cover the basic options: a number of other options can be found in the README.formatdb file.By default, format db produces 3 files with the same base name but different extensions.  For example, ath.nhr, ath.nsq, and ath.nin.  The base name is “ath”, and these are nucleic acid files because the first letter of the extensions is n (it would be p for protein files).The three necessary parameters for formatdb are:-i input data file (containing one or more sequences in FASTA format)-n output file base name (if this parameter is not set, the input file name is used as base)-p type of file: T for protein, F for nucleic acidThe –o option produces another set of files used for indexing.  This of course uses more space.  I haven’t found it very useful. Basic syntax on the command line:	formatdb –i my_input_genes.txt –p F –n my_genesThis takes a FASTA file containing nucleic acid sequences and creates the database files my_genes.nhr, my_genes.nsq, and my_genes.nin.
