# Supplementary material for: Genome-Wide Detection of Spontaneous Chromosomal Rearrangements in Bacteria
Source: PLoS One. 2012 Aug 3;7(8):e42639. doi: 10.1371/journal.pone.0042639 (PMC3411829; doi:10.1371/journal.pone.0042639)
Supplement: Scripts S1 — Custom perl scripts used split-read mapping filtering, sequencing quality analysis, and generation of in silico chimeric reads. (ZIP) [file pone.0042639.s010.zip › user mannual.rtf]

Block 1 (Initial Screening)Input filestracesequences.fasta (454sequencingdata)genomesequence.fnaProgrammesformatdbblastallPerl scriptsformatduplication_first.plformateduplication_second.plduplicationdetection.plsortduplicationdetectionresult.plannoteduplication.plformatgenomesequence.plreversecomplement.plProcedures (delete the first line in the new output file if it is a blank line)1. make a local database using formatdb 2. ./blastall -p blastn -i trace_sequences.fasta -d genome -F F -e .0001 -o genome_directblast.txt3. perl formatduplication_first.pl genome_directblast.txt > genome_directblast_firstformated.txt4. perl formateduplication_second.pl genome_directblast_firstformated.txt > genome_directblast_secondformated.txt5. perl duplicationdetection.pl genome_directblast_secondformated.txt > genome_directblast_duplicationdetection.txt6. perl sortduplicationdetectionresult.pl genome_directblast_duplicationdetection.txt > genome_directblast_duplicationdetection_sorted.txt (ignore minus homology reads)7. perl annoteduplication.pl genome_directblast_duplicationdetection_sorted.txt > genome_directblast_duplicationdetection_sorted_annotated.txt (reset the value for genome size )8. perl formatgenomesequence.pl genomesequence.fna > formated_genome.txt (reset the value for genome name)9. perl reversecomplement.pl formated_genome.txt > reversecomplement_genome.txt (reset the value for genome name)Block 2 (analyze the results from initial screening to exclude the false hits possibly due to sequencing errors)Input filess: genome_directblast_duplicationdetection_sorted_annotated.txttracesequences.fastaformated_genome.txtreversecomplement_genome.txt Perl scriptschangereadsformat.pladdreadlengthandread.plformatjunctionsequenceorder.plfilterandgetsequence.pl (reset $chrsize for different genome)getjunctionsequence.plstringent_gettruejunctions.plProcedures:1. perl changereadsformat.pl tracesequences.fasta > genome_tracesequences_formated.txt2. perl addreadlengthandread.pl genome_directblast_duplicationdetection_sorted_annotated.txt  genome_tracesequences_formated.txt > traces_directblast_sorted_annotated_withtracesequence.txt    (add the length of each trace sequence containing candidate junction sequence and the sequence)3. perl formatjunctionsequenceorder.pl traces_directblast_sorted_annotated_withtracesequence.txt > traces_directblast_sorted_annotated_withtracesequence_formated.txt    (format the order of four position number in the trace sequence $a[2]  $a[4]  $a[7]  $a[9] to make  $a[2]  < $a[7])4. perl filterandgetsequence.pl traces_directblast_sorted_annotated_withtracesequence_formated.txt   formated_genome.txt   reversecomplement_genome.txt > traces_directblast_afterfiltered.txt  (set chrSize)(if the both of overhangs are more than overlap_threshold[15], regarded as true; if any of overhangs are less  than boundary_threshold[5], regarded as false, unless cover the whole read (regarded as true but marked with * ); if any of overhangs are between 4 and 15, print out the sequence in read, matched sequence and unmatched sequence)5 check manually, and make all confirmed trace number in the file traces_directblast_truejunctions_name.txt6. perl gettruejunctionsequence.pl traces_directblast_truejunctions_name.txt traces_directblast_duplicationdetection_sorted_annotated.txt > traces_directblast_truejunctions.txt    stringent_gettruejunctions.pl regard all overhangs less than boundary_threshold as false no matter if marked with * or notBlock3 (screen junction-spanning reads by quality scores first, and then perform statistical analysis )Firstly divide all candidate junctions into two parts according to the sign (+ or -) of junction homology (<0 homology indicate non-template sequences at junctions)For all reads with >=0 bp junction homology1. perl clearandstringent.pl junctiondetection_annotated.txt > junctions_cleaned.txt  #get rid of pre-existed junctions and reads with less than 5bp overhanges on both sides)2. perl averagequalityscore_+-5bponeithersideofoverlappingsequence.pl junctions_cleaned.txt formatedqualityscorefile > junctions_cleaned_withaveragequalityscore+-5bponeitherside.txt  3. perl averagequalityscore_overlappingsequences.pl  junctions_cleaned.txt formatedqualityscorefile > junctions_cleaned_withaveragequalityscoresforoverlap.txt4. perl removecommonjunctions.pl junctions_cleaned.txt junctions_cleanedcopy.txt > junctions_cleaned_commonjunctionsremoved.txt5. perl getreadnamesandthreequalityscore.pl junctions_cleaned_commonjuctionsremoved.txt junctions_cleaned_withaveragequalityscoresforoverlap.txt junctions_cleaned_withaveragequalityscore+-5bponeitherside.txt > junctions_namesandthreequalityscores # up5,down5,overlap6. perl samplequalityscorerandomwithoverlaplength.pl junctions_cleaned_commonjunctionsremoved.txt formatedqualityscorefile > randomoverlaplengthqualityscore.txt7. perl samplequalityscorerandom_5bp.pl formatedqualityscorefile > random5bpqualityscore.txt8. formateaveragequalityscoreforeachreadincsvformat.pl #use this script to format .txt into .csv file which can be used for R9. compare the quality score data and simulated quality score by using Mann Whitney U test10.  perl extractsingletonswithmorethan20qualityscore.pl junctions_commonjunctionsremoved.txt junctions_namesandthreequalityscores.txt > junctions_singletons_qualified.txt11. perl  findcommon.pl junctions.txt junctionscopy.txt > commonjunctions.txt ( combine with quality score to confirm them)12 the file junctions_singletons_qualified can be used for mirohomology distribution analysisFor non-template sequences containing reads1. extract all nontemplate candidates (<10bp homology)2. perl qualityscores_+10_nontemplate_-10.pl insertioncandidates.txt formatedqualityscorefile > insertioncandidates_withqualityscorefor+10overlap-10region.txt3. perl screennontempaltejunctionsbasedonqualityscores20+.pl inserstioncandidates.txt insertioncandidates_withqualityscorefor+10overlap-10region.txt > nontemplatejunctions_afterscreened.txtStatistics(simulation)Input filesformated_genome.txtreversecomplement_genome.txtPerl scriptssimulationforrandomsequence.plsimulationforhybridsequence.plProceduresperl simulationforrandomsequence.pl formated_genome.txt reversecomplement_genome.txt > perl simulationforhybridsequence.pl formated_genome.txt reversecomplement_genome.txt >(reset genomesize, lengthofsequence and numberofsimulatedsequence)
